# Sinogram: A Valuable Diagnostic Tool for an Adult Male With Discharging Heel Ulcer and Suspected Calcaneal Osteomyelitis

**DOI:** 10.7759/cureus.43670

**Published:** 2023-08-17

**Authors:** Pokhraj P Suthar, Raman Shingade, Lavanya Chhetri, Divya Saini, Avin Kounsal

**Affiliations:** 1 Department of Diagnostic Radiology and Nuclear Medicine, Rush University Medical Center, Chicago, USA; 2 Department of Radiology, Nidan Diagnostic and Research Center, Bhubaneswar, IND; 3 Department of Clinical Nutrition, Rush University Medical Center, Chicago, USA; 4 Department of Public Health, Johns Hopkins Bloomberg School of Public Health, Baltimore, USA

**Keywords:** imaging, heel ulcer, adult, sinogram, calcaneal osteomyelitis

## Abstract

This report highlights the clinical significance of a sinogram in diagnosing osteomyelitis in resource-poor areas. We report a case in which a sinogram was used successfully for the diagnosis of calcaneal osteomyelitis. A 25-year-old male patient sought medical attention for persistent pain in the right ankle joint and heel, accompanied by a discharging ulcer over the right heel. He had a history of foot trauma involving vegetative matter sustained during a farming injury one month prior to the onset of symptoms. An ankle radiograph revealed an osteolytic lesion involving the calcaneum, prompting further investigation with a sinogram, which indicated a subcutaneous sinus tract with intramedullary extension into the calcaneum. Despite the clinical necessity for a magnetic resonance imaging (MRI) or computed tomography (CT) evaluation to precisely assess the lesion's extent and aid in formulating an appropriate treatment plan, the patient faced significant financial constraints that hindered him from undergoing the essential imaging procedure.

## Introduction

Osteomyelitis, characterized by the inflammation of the bone and bone marrow, was first described by Nelaton in 1844, deriving its name from the Greek words "osteon" (bone), "myelo" (marrow), and "itis" (inflammation) [1]. The introduction of antibiotics led to a significant global decline in osteomyelitis cases. However, recent trends have shown an alarming increase in incidences among specific vulnerable populations, including immunosuppressed individuals, alcoholics, newborns, and drug addicts [2]. Imaging findings of a sinogram in chronic osteomyelitis include retrograde contrast opacification of a sinus tract, which outlines the sinus's path and extent and identifies associated bone cavities or abscesses. The sinogram proved a valuable diagnostic tool, revealing critical insights into the extent of the infection and its intramedullary involvement, aiding in appropriate management decisions for the patient. Access to advanced diagnostic imaging, such as magnetic resonance imaging (MRI)/computed tomography (CT), may be limited in resource-constrained regions. This case report emphasizes the importance of employing a sinogram as an alternative imaging modality for diagnosing suspected osteomyelitis in underprivileged populations.

## Case presentation

**Figure 1:**
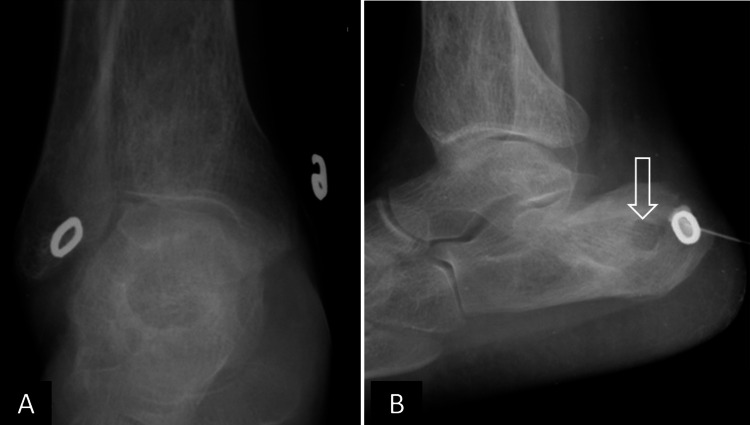
Initial sinogram X-ray of the right ankle. (A) Anteroposterior and (B) lateral views show a well-defined, osteolytic lesion in the calcaneum with subtle peripheral sclerosis (open white arrowhead in B). Minimal periosteal reaction is seen in the calcaneum. Sinogram external marker and needle are seen in B.

**Figure 2:**
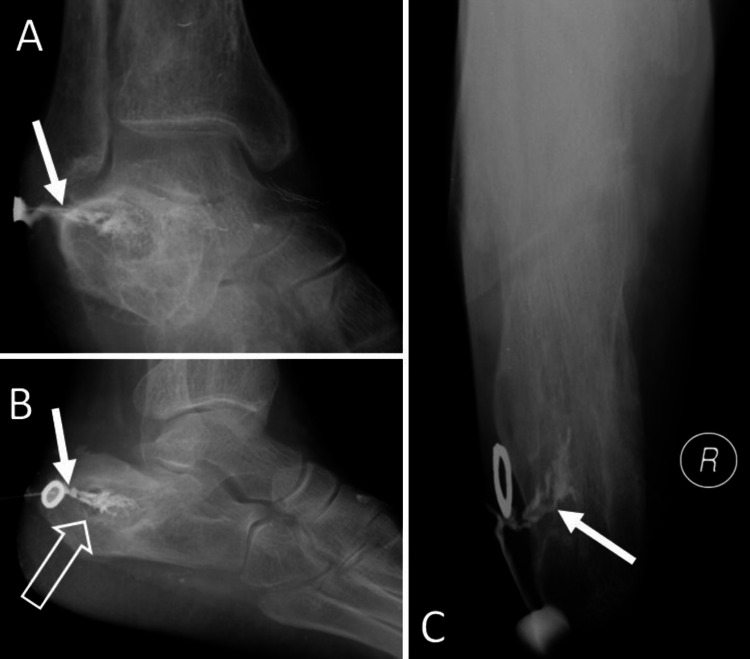
Sinogram after contrast injection reveals well-opacified sinus tract extending from skin surface and subcutaneous tissue to the calcaneum (solid white arrows in A-C). A well-defined, osteolytic lesion in the calcaneum with subtle peripheral sclerosis (open white arrow in B).

**Figure 3:**
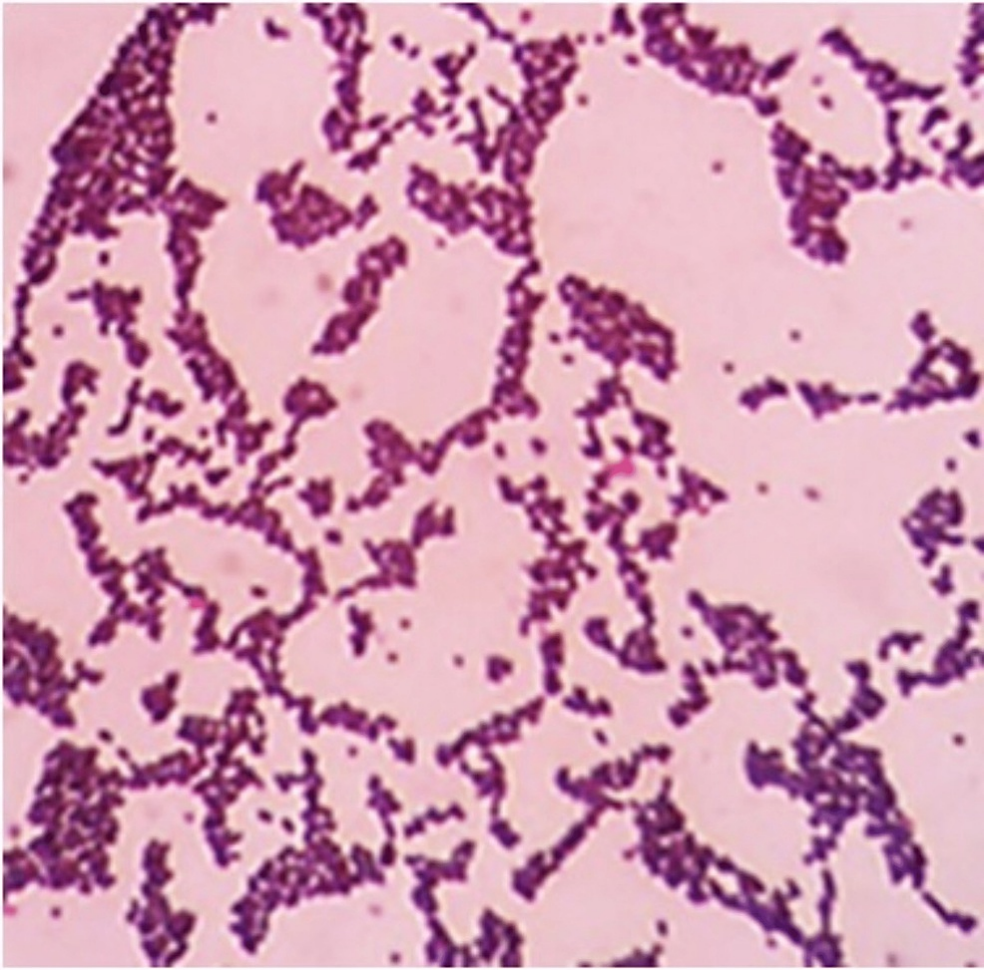
Gram staining of the sinus discharge. The Gram stain of the specimen showed gram-positive cocci in clusters, suggestive of* Staphylococcus aureus.*

A 25-year-old male farmer from a rural area in the western part of India, with no comorbidities, presented with complaints of pain in his right heel and ankle, as well as a discharging ulcer on the right heel that had persisted for over a month. His symptoms were preceded by foot trauma involving vegetative material sustained while farming, a month prior to his presentation. Additionally, the patient experienced low-grade fever and malaise but denied any history of tuberculosis, diabetes, addiction, or steroid therapy. The patient was febrile on examination, while the rest of the vital signs and systemic examination were within normal limits. There was a 3×2 cm well-marginated ulcer on the right heel without bleeding points. The surrounding tissue appeared mildly inflamed and tender. The ulcer progressively increased in size with associated pus discharge. The patient was unable to bear weight on that heel. A complete blood count revealed a neutrophil-predominant mild leukocytosis (total white blood cell {WBC}=11,900/mm^3^), while the remaining blood counts were within normal ranges. The erythrocyte sedimentation rate (ESR) was elevated at 27 mm/hour. Serum glucose, electrolytes, and chest X-ray were all within normal limits. There was no significant past history of nonhealing ulcers. Enzyme-linked immunosorbent assay (ELISA) testing for HIV was negative. X-ray of the right ankle revealed a well-defined osteolytic lesion in the calcaneum with a radiolucent tract extending from the lesion. A minimal periosteal reaction involving the calcaneum was also observed (Figure 1). Although an MRI/CT evaluation was clinically necessary to accurately assess the extent of the lesion and assist in formulating an appropriate treatment plan, the patient encountered significant financial constraints that prevented him from undergoing the essential imaging procedure. As an alternative, sinogram imaging was conducted using diluted iodinated contrast and various views to provide valuable insights into the condition. Sinogram imaging revealed a well-opacified sinus tract extending from the skin surface and subcutaneous tissue to the calcaneum (Figure 2). A microscopic analysis of the sinus discharge was performed, and it tested positive for *Staphylococcus aureus* (Figure 3).

The patient underwent treatment, which included sinus debridement, followed by a five-day course of intravenous cefuroxime. Subsequently, the patient was switched to oral cefadroxil, for the treatment of methicillin-sensitive *Staphylococcus aureus* (MSSA). Subsequently, on the 10th day of hospitalization, the patient was discharged with significant relief of symptoms. However, it is unfortunate that the patient was lost to follow-up, which limited the ability to conduct further imaging evaluations and track the long-term outcome.

## Discussion

Osteomyelitis, a bone infection, is an acute or chronic inflammatory condition affecting the bone and its associated structures. It arises from infection by pyogenic organisms such as bacteria, fungi, and mycobacteria [2]. Osteomyelitis can be classified into four types based on its pathogenesis: hematogenous, post-traumatic, true contiguous spread, or iatrogenic. Among these, hematogenous osteomyelitis is the most common form. While osteomyelitis can affect individuals of all ages, it is more commonly observed in those under 20 or over 50 years of age [3]. In hematogenous osteomyelitis, children often exhibit infections in long bones, especially the metaphyses of the femur and tibia. In contrast, adults frequently show osteomyelitis in the vertebrae, primarily in the lumbar region. Contiguous focus osteomyelitis arises from adjacent infections or surgical interventions, commonly affecting foot bones such as the calcaneus, particularly in patients with diabetic foot ulcers. Osteomyelitis can also occur near joint prosthetics or in bones such as the tibia following open fractures. The site of osteomyelitis due to direct inoculation often corresponds to the trauma or surgical site [3,4]. However, osteomyelitis of the calcaneum is a rare condition, accounting for approximately 5%-8% of all cases of osteomyelitis [5]. *Staphylococcus aureus* is the most commonly implicated organism, responsible for around 90% of all cases of osteomyelitis [6]. Other causative organisms include* Haemophilus influenzae*, *Streptococcus pneumoniae*, *Mycobacterium tuberculosis*, and *Pseudomonas*.

The clinical presentations of calcaneal osteomyelitis can vary significantly based on age groups. Common symptoms include fever, malaise, edema, erythema, and pain in the affected area [7]. Many patients also experience difficulty in bearing weight. In chronic and subacute cases, systemic features are usually absent. A characteristic posture in which patients avoid contact of the heel to the bed has been reported in some studies as a notable feature. Early diagnosis is crucial to prevent prolonged morbidity. Laboratory investigations, such as erythrocyte sedimentation rate (ESR) and C-reactive proteins, are often elevated but may not strongly indicate infection [8]. WBC counts and serum alkaline phosphatase may also show elevation.

Radiographic manifestations may take 1-2 weeks to appear after clinical symptoms. They are, however, used to rule out other potential causes of symptoms, such as fractures or metastasis. Initial X-rays are usually normal, but later, common findings include local soft tissue swelling, changes in bone density (osteopenia), periosteal elevation, and the formation of an involucrum. In chronic cases, multiple cloacae pass through the involucrum, and soft tissue may open on the skin. Furthermore, a thickened irregular cortex and dense sclerotic bone surrounding focal lytic areas may be observed [1].

Computed tomography (CT) and magnetic resonance imaging (MRI) exhibit higher sensitivity than radiography in identifying osteomyelitis-related changes. CT effectively reveals early changes in cortical bone and soft tissues, while MRI is adept at detecting early changes in the bone marrow and soft tissues. The MRI stands out with the highest combined sensitivity and specificity, ranging from 78% to 90% and 60% to 90%, respectively, for detecting osteomyelitis [9]. T1-weighted (T1W) sequences in MRI offer excellent anatomical detail, allowing clear visualization of the medulla, cortex, periosteum, and soft tissues. In T1W images, the fluid appears dark with low signal intensity, abscesses show low to intermediate signal intensity, and fat exhibits high signal intensity. Fluid-sensitive sequences, including T2-weighted (T2W), fat-suppressed (FS), and short-tau inversion recovery (STIR) sequences, are valuable for detecting infection and inflammation, which leads to an increase in tissue fluid content. However, the use of MRI and CT may be limited in cases where surgical hardware is present. The presence of metallic implants and other surgical materials can cause artifacts and interfere with the image quality, making obtaining precise and accurate images in such situations challenging. Alternative imaging approaches or careful interpretation of the available images may be necessary in these cases.

Nuclear medicine imaging detects osteomyelitis 10-14 days earlier than plain radiographs. Agents such as technetium-99m methylene diphosphonate (^99m^Tc-MDP), gallium-67 citrate, and indium-111-labeled white blood cells show high sensitivity but low specificity, challenging differentiation from other conditions [10]. Nuclear medicine scans are helpful adjunctive studies when pathologic or postsurgical changes alter X-rays. Compared to three-phase bone scintigraphy/single-photon emission computed tomography (SPECT), SPECT/CT offers improved diagnostic precision in assessing osteomyelitis. Beyond potentially reshaping patient diagnosis and treatment plans, it provides enhanced image clarity and resolution, capturing even subtle activity increments. Additionally, SPECT/CT is invaluable in identifying hardware infections.

Sinogram imaging offers a cost-effective alternative to diagnosing osteomyelitis. It has proven to be a valuable diagnostic tool, providing critical insights into the extent of the infection and its involvement within the bone marrow, which helps guide appropriate management decisions for the patient. The process involves inserting a small, flexible catheter into a cutaneous opening, followed by retrograde injection of contrast material to opacify the sinus tract. This enables the visualization and mapping of the sinus tract's course and extent, including its potential connections to nearby structures. To further enhance the visualization and delineation of the sinus tracts, sinography can be combined with CT imaging, offering a comprehensive and detailed evaluation of the affected area [11]. To establish a conclusive diagnosis, a bone biopsy is essential as it allows for the identification of the causative organism.

Managing calcaneal osteomyelitis requires a collaborative and multidisciplinary approach involving the expertise of orthopedic surgeons, plastic surgeons, and infectious disease physicians. A commonly employed broad-spectrum empiric antibiotic regimen, effective against both gram-positive and gram-negative organisms, including methicillin-resistant *Staphylococcus aureus* (MRSA), consists of intravenous vancomycin in combination with a third-generation cephalosporin or a beta-lactam/beta-lactamase inhibitor combination. As soon as sensitivity data becomes available, antibiotic therapy should be adjusted to provide targeted coverage for susceptible organisms [2].

## Conclusions

Osteomyelitis of the calcaneum is relatively uncommon, highlighting the need for early detection. Timely recognition and appropriate imaging are vital for effective management and improved outcomes, especially in patients with heel and ankle pain and a history of foot trauma. Sinography, in conjunction with plain radiography, is a dependable diagnostic method, particularly in areas with limited MRI/CT accessibility. Bone biopsy confirms the causative organism and guides antibiotic therapy. A comprehensive, multidisciplinary approach is essential for successful management. This case emphasizes the significance of sinograms in MRI/CT-inaccessible areas.
